# Tietze Syndrome

**DOI:** 10.31138/mjr.31.2.224

**Published:** 2020-04-24

**Authors:** Giuseppe Mettola, Carlo Perricone

**Affiliations:** 1 Reumatologia, Dipartimento di Scienze Cliniche, Internistiche, Anestesiologiche e Cardiovascolari, Sapienza Università di Roma, Italy; 2 Reumatologia, Dipartimento di Medicina, University of Perugia, Italy

**Keywords:** Tietze, spondyloarthritis, cervicobrachial syndrome, sterno-clavicular joint, magnetic resonance, costochondritis

A 43-year-old man (C.L.) presented with a 7-year history of stiffness of the cervical spine with symptoms in the shoulder girdle and upper extremity, especially on the right side, that he referred related to post-traumatic sport-associated events. For this purpose, he used analgesics with some temporary benefit. After falling from the motorbike, he had broken coccyx and medial twin muscle. He also referred cubital tunnel syndrome, slipped disc in L5-S1, pain to the right foot and ankle. Conventional radiography demonstrated cortical bone irregularities due to osteoarthritis on first right metatarsal-phalangeal joint.

**Figure 1.**
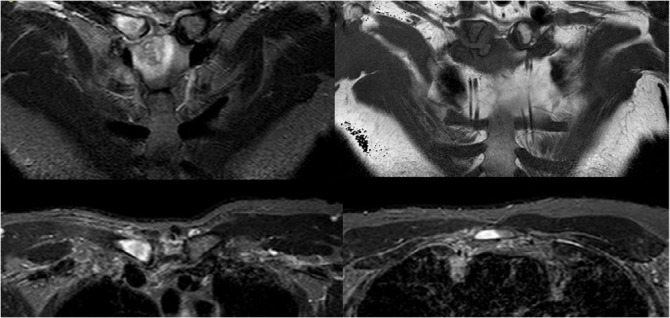
Magnetic resonance imaging showed mild synovial reaction and intra-articular effusion with prominent bone marrow oedema.

After 2 months, cervicobrachial syndrome on the right side still persisted, the patient referred recurrent cystitis, and noted a swelling of the right sterno-clavicular joint. Magnetic resonance imaging showed synovial reaction and intra-articular effusion with bone marrow oedema of the right sterno-clavicular joint (*Figure 1*). Magnetic resonance is an excellent technique to show cartilaginous, joints and bone abnormalities, therefore it represents the elective method in the investigation of Tietze syndrome. It has been employed in cases of chest wall pain following thoracic trauma, spondyloarthropathies, septic arthritis and malignant tumours that may mimic Tietze syndrome. It is also important to discern a clear costochondritis from Tietze syndrome. The first is more common and affects people older than 40 years. It affects more than one costochondral junction (especially from second to fifth) and local swelling is absent. The second one is rare and strikes younger individuals. It affects one costochondral junction (the second or the third in about 70 percent of patients), jointed to local swelling. It is also reported that females are diagnosed with the disease more often than males by a 2:1 ratio. This patient is an uncommon case. He is a 43-year-old man, but the imaging features are typical of Tietze syndrome. Since the disease is often spontaneously resolving, it was suggested the usage of coxib (etoricoxib 90 mg/day for 3 months) and physical therapy. The patient after 6 months is treated with etorixib 60 mg PRN, with no more symptoms have been referred so far.

